# Successful management of severe bronchial asthma exacerbated by anti‐PD‐L1 treatment: A report of two cases

**DOI:** 10.1002/rcr2.868

**Published:** 2021-10-24

**Authors:** Toshiyuki Sumi, Yuta Nagahisa, Keigo Matsuura, Motoki Sekikawa, Yuichi Yamada, Hisashi Nakata, Hirofumi Chiba

**Affiliations:** ^1^ Department of Pulmonary Medicine Hakodate Goryoukaku Hospital Hakodate Japan; ^2^ Department of Respiratory Medicine and Allergology Sapporo Medical University School of Medicine Sapporo Japan

**Keywords:** immune checkpoint inhibitor, irAE, mepolizumab, severe asthma

## Abstract

Immune checkpoint inhibitors (ICIs) have been used for various carcinomas. However, immune‐related adverse events have been observed. There have been few reports of treatment with biologics for severe bronchial asthma induced by ICI; therefore, their efficacy is unknown. We report two cases of severe bronchial asthma requiring systemic steroid administration while using anti‐programmed death‐ligand 1 (PD‐L1) antibody for advanced non‐small‐cell lung cancer. The anti‐interleukin‐5 antibody, mepolizumab, was introduced, resulting in the discontinuation of systemic prednisolone and good asthma control. These reports suggest that treatment with biologics may be effective in severe cases of poorly controlled bronchial asthma during ICI therapy.

## Graphical abstract

We report two cases of severe bronchial asthma requiring systemic steroid administration while using anti‐programmed death‐ligand 1 (PD‐L1) antibody for advanced non‐small cell lung cancer. In each case, mepolizumab was introduced, resulting in discontinuation of systemic prednisolone and good control of asthma.

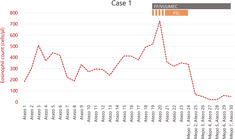

## INTRODUCTION

Immune checkpoint inhibitors (ICIs) have been used for various carcinomas and have become the mainstay of chemotherapy. Anti‐programmed death‐1 (PD‐1) protein and anti‐programmed death‐ligand 1 (PD‐L1) antibodies activate anti‐tumour immunity by inhibiting the binding of PD‐L1 of tumour cells to PD‐1 of immunocytes.^1^ However, various immune‐related adverse events (irAEs) are observed. Among them, eosinophil‐induced irAEs are rare, accounting for about 2.9%–3.3%.^2^, ^3^ Only a few cases of bronchial asthma development and severity have been reported as allergy‐related irAE associated with ICI.^4^, ^5^, ^6^, ^7^ The current understanding of these irAEs is limited given the limited availability of cases.

Steroids are the main treatment for irAE and do not influence the prognosis of lung cancer.^8^ Management of irAE with appropriate steroid administration is also necessary to allow the ICI treatment to continue.^9^ On the other hand, in recent years, it has been reported that in patients with asthma, the risk of side effects such as osteoporosis, hypertension and gastrointestinal ulcers increases as the frequency of steroid administration increases, even if the administration is short term.^10^ Mepolizumab, an anti‐human interleukin‐5 (IL‐5) antibody, is effective against severe bronchial asthma, reducing the use of steroids and suppressing asthma exacerbations.^11^, ^12^ Mepolizumab is also approved for eosinophilic granulomatosis with polyangiitis (EGPA) and severe paediatric bronchial asthma.^13^, ^14^ In severe bronchial asthma, discontinuation of treatment with mepolizumab significantly increases exacerbations and worsens asthma control; thus, its continuation is advisable whenever possible.^15^ Mepolizumab inhibits the eosinophil proliferative effect of IL‐5 by specifically binding to IL‐5 and inhibiting its binding to the IL‐5 receptor alpha chain expressed on the cell surface of eosinophils. There have been few reports of treatment with mepolizumab for severe bronchial asthma induced by ICI^5^ and its efficacy is still unknown.

We report two cases of severe bronchial asthma requiring systemic steroid administration while using anti‐PD‐L1 antibody for advanced non‐small cell lung cancer. In each case, mepolizumab was introduced, resulting in discontinuation of systemic prednisolone (PSL) and good control of asthma.

## CASE REPORT

### Case 1

**FIGURE 1:**
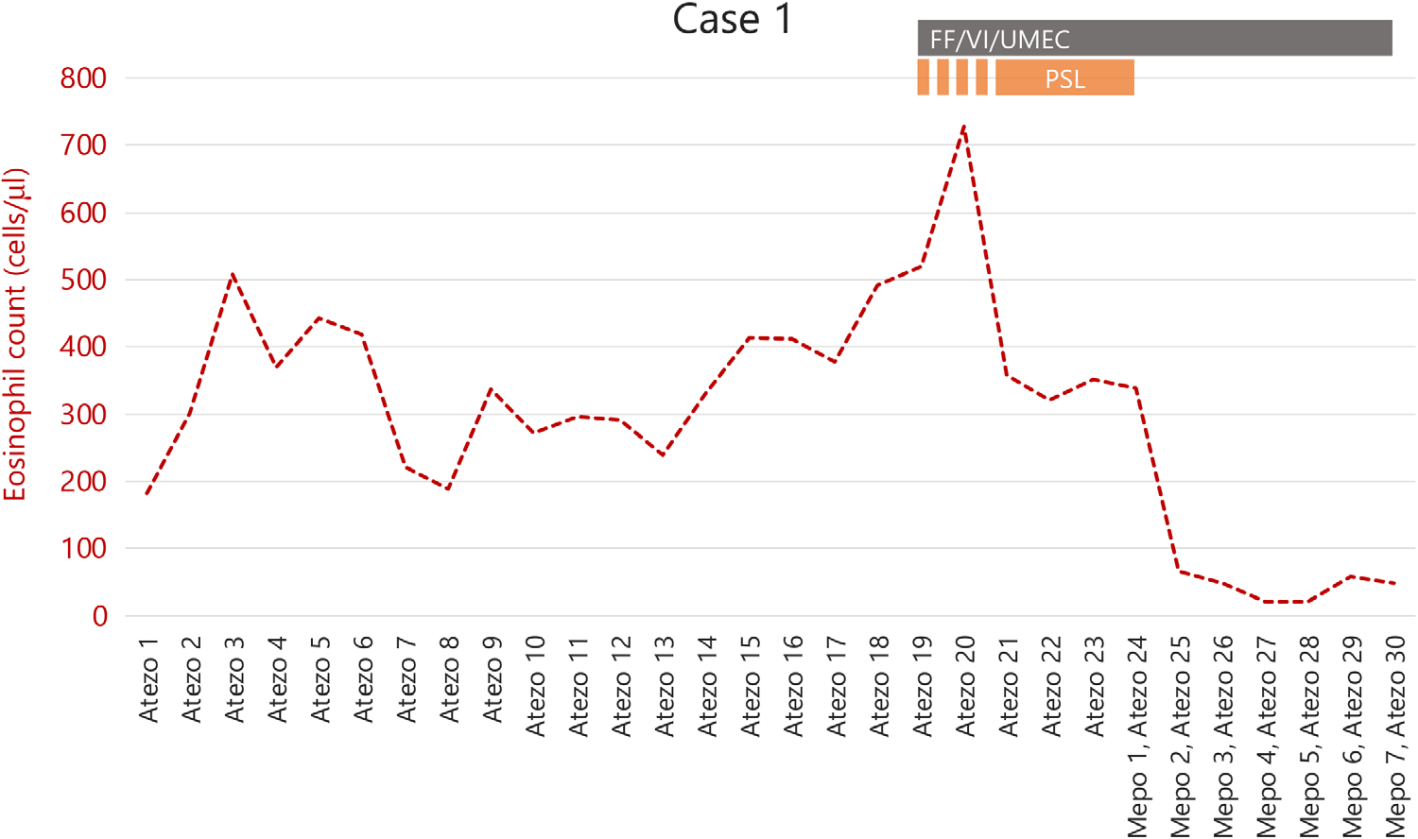
Clinical course of case 1. Triple therapy and PSL administered with Atezo from course 19 show no significant improvement. Mepo was additionally administered with Atezo from course 24, with an immediate response. Atezo, atezolizumab; FF/VI/UMEC, fluticasone furanecarboxylate, vilanterol, umeclidinium; Mepo, mepolizumab; PSL, prednisolone

The patient was a 70‐year‐old man with a 10 pack‐year history of smoking. He had a history of allergic rhinitis and bronchial asthma, but his symptoms were stable without use of inhaled corticosteroids (ICS). He was diagnosed with squamous cell carcinoma of the lung cT4N3M1a stage IVA. The epidermal growth factor receptor mutation was negative, with PD‐L1 < 1%, and he received two courses of cisplatin (60 mg/m^2^, day 1) and tegafur/gimeracil/oteracil (TS‐1) (120 mg/body, days 1–14) as the first‐line treatment. No granulocyte colony‐stimulating factor (GCSF) drugs were used during the initial chemotherapy course, and the eosinophil count did not exceed 300. The primary tumour was enlarged, and the diagnostic evaluation was progressive disease (PD). Atezolizumab, an anti‐PD‐L1 antibody (1500 mg/body, every 3 weeks), was started as the second‐line therapy. The tumour shrank, and the best effect was a partial response (PR). However, from the 19th course, daily coughing, wheezing and nocturnal symptoms appeared; therefore, fluticasone furanecarboxylate (200 μg)/umeclidinium bromide (62.5 μg)/vilanterol (25 μg) and a leukotriene receptor antagonist (LTRA) were administered. However, the eosinophil count, which had been elevated before treatment (Figure 1), rose further (from approximately 580 to 710), and 30 mg PSL was administered frequently as a burst dose and continuous PSL was required. Respiratory function tests showed forced expiratory volume in 1s (FEV_1_) of 1.59 L, FEV_1_/forced vital capacity (FVC) of 64.37% and fractional exhaled nitric oxide (FeNO) of 78 ppb (normal range: 15–37 ppb). After a diagnosis of severe asthma and initiation of mepolizumab (100 mg/body, every 4 weeks), these symptoms resolved. At the fourth dose, the symptoms were almost completely resolved and the respiratory function tests showed FEV_1_ of 1.88 L, FEV_1_/FVC of 54.49% and FeNO of 25 ppb. Atezolizumab was continued with mepolizumab until the 30th course of PD and is ongoing.

### Case 2

**FIGURE 2:**
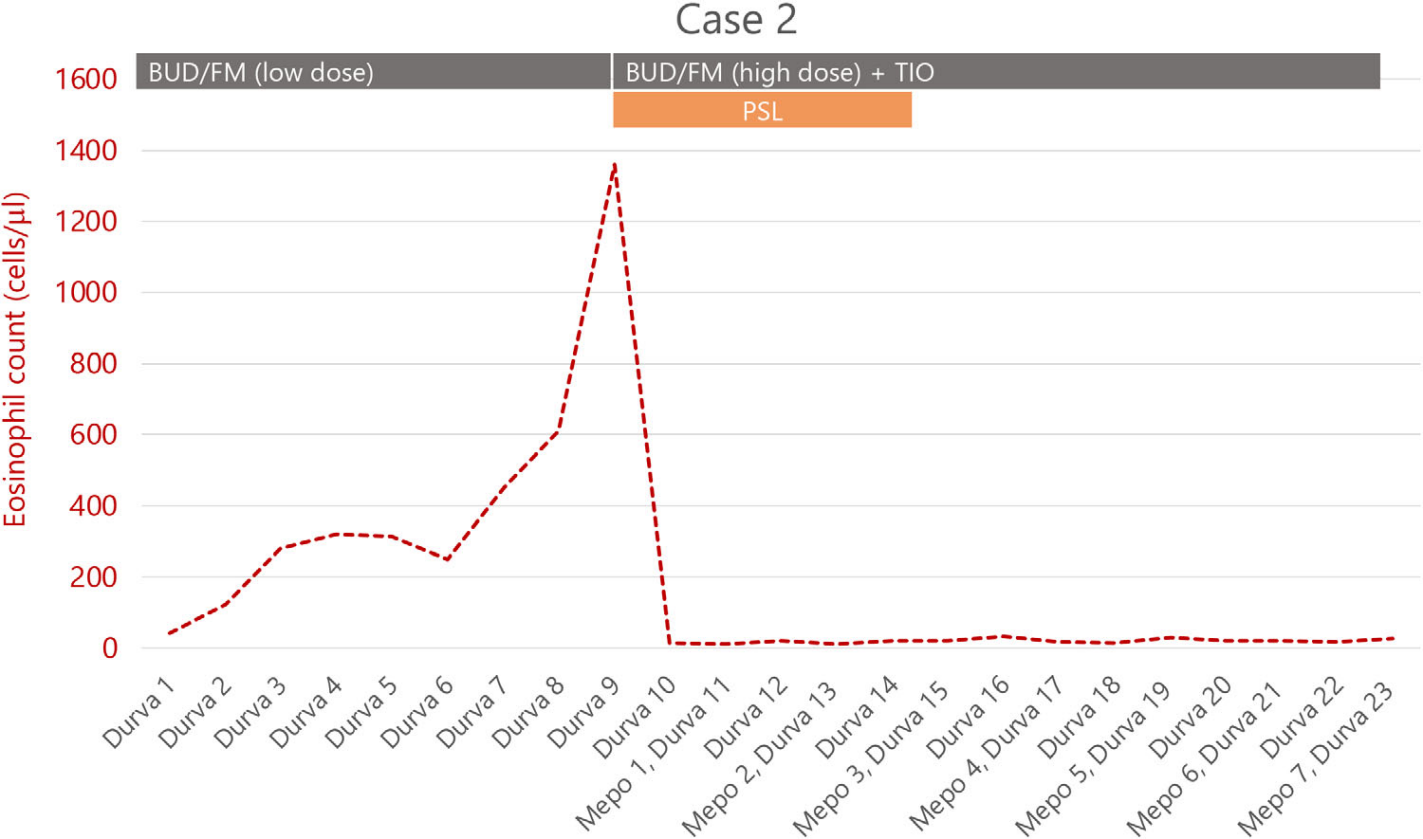
Clinical course of case 2. High‐dose corticosteroid/beta‐agonist/muscarinic antagonist was supplemented with Mepo to produce the desired response. BUD/FM, budesonide, formoterol; Durva, durvalumab; Mepo, mepolizumab; PSL, prednisolone; TIO, tiotropium

The patient was a 65‐year‐old man with a 15 pack‐year history of smoking. He had a history of diabetes mellitus and bronchial asthma, which were well controlled with oral hypoglycaemic agents (linagliptin 5 mg) and low doses of budesonide (320 μg)/formoterol (9 μg). He was diagnosed with squamous cell carcinoma of the lung cT4N1M0 stage IIIA, with PD‐L1 1%, and underwent chemoradiotherapy. Chemotherapy consisted of cisplatin (day 1) and TS‐1 (days 1–14) every 4 weeks for 2 cycles. Once‐daily thoracic radiotherapy was administered at a total dose of 60 Gy, 5 days a week, in 30 fractions. No GCSF agents were used during chemoradiation therapy, and the eosinophil count never exceeded 300. As the treatment effect was PR, durvalumab was then administered as maintenance therapy. After the start of durvalumab, the number of eosinophils in the peripheral blood gradually increased from 50 to approximately 300 (Figure 2), and the patient experienced asthma flare‐up in the ninth course. Daily oral steroids (PSL 10 mg/day) were administered, ICS was increased to a higher dose of budesonide (960 μg)/formoterol (27 μg) and tiotropium and an LTRA were added. Despite this, asthma control was inadequate, and the steroids elevated blood glucose levels to 400 mg/dl. Oral steroids reduced the eosinophil levels, but symptoms persisted. The patient was diagnosed with severe bronchial asthma and started on mepolizumab in the 11th course. Before starting mepolizumab, respiratory function tests showed FEV_1_ of 1.44 L, FEV_1_/FVC of 50.17% and FeNO of 47 ppb. Asthma control improved from the second dose of mepolizumab, and respiratory function tests at the fourth dose of mepolizumab showed FEV_1_ of 1.57 L, FEV_1_/FVC of 43.85% and FeNO of 16 ppb. At the third dose of mepolizumab, oral steroids could be discontinued, and blood glucose levels stabilized between 100 and 150 mg/dl. He completed the durvalumab maintenance therapy while continuing with mepolizumab. For the past year, there has been no tumour recurrence or asthma exacerbation.

## DISCUSSION

We encountered two cases of severe bronchial asthma, requiring systemic steroids, that was induced by anti‐PD‐L1 antibody treatment in lung cancer patients with a history of bronchial asthma. We achieved good control of bronchial asthma symptoms by using an anti‐IL‐5 antibody and were able to discontinue systemic steroids and continue ICIs.

In the KEYNOTE‐002, 006, 010, 024, 041, 042, 045, 048, 054, 087, 158, 164 (Cohort A), 177, 181, 189, 355, 407 and 426 trials, bronchial asthma as an adverse drug reaction was observed in two of 5339 patients and there were no serious cases (Reference from the drug interview form for pembrolizumab in Japan). Bronchial asthma is thus a very rare side effect of the use of ICIs. Nevertheless, similar to the cases presented, there are several reports of bronchial asthma onset and exacerbation during the use of ICIs.^4^, ^5^, ^6^, ^7^ The relationship between PD‐1 and PD‐L1/PD‐L2 in bronchial asthma has been reported recently, and airway hyperresponsiveness is increased when anti‐PD‐1 and anti‐PD‐L1 antibodies are administered to mice.^16^ In addition, there is a negative correlation between PD‐1 expression on CD4‐positive T cells and total IgE levels and specific IgE antibodies in asthma patients.^17^ In other allergic diseases, induction of PD‐L1 in B cells of patients with cedar pollinosis suppresses the production of IL‐5 and IL‐13 in CD4‐positive T cells.^18^ In patients with allergic rhinitis, there is a negative correlation between the amount of soluble PD‐L1 and the peripheral blood eosinophil count and the severity of allergic rhinitis.^19^ Although the mechanism of severe bronchial asthma during ICI use has not been elucidated, it might be speculated that the exacerbation of bronchial asthma, in this case, was mediated by enhancement of Th2‐type inflammation by the anti‐PD‐L1 antibody.

On the other hand, eosinophils are also components of the tumour immune microenvironment. They are a source of anti‐tumour (e.g., tumour necrosis factor‐alpha) and pro‐tumour (e.g., vascular endothelial growth factor) molecules. Eosinophils play an anti‐tumour role in some neoplasms (e.g., melanoma, gastric cancer and colorectal cancer), whereas they are associated with poor prognosis in others (e.g., diffuse large B‐cell lymphoma and lung adenocarcinoma).^20^ In addition, a retrospective study reported that eosinophilia during the use of ICIs correlates with treatment response.^21^ In the same study, when ICIs were used for solid tumours, including non‐small cell lung cancer, 22% of the patients developed hypereosinophilia >500/μl. The higher the eosinophil count, the better the disease control, but also greater the chance of developing toxicity. Thus, eosinophils might be closely related to tumour immunity, and it is unclear whether extreme reduction in eosinophils by biologics will exacerbate tumours; therefore, biologics should be administered with caution. Based on this perspective, we used anti‐IL‐5 rather than anti‐IL‐5αR antibodies, which reduce the eosinophil count to zero by antibody‐dependent cellular cytotoxicity activity, for the present cases.

In bronchial asthma, exacerbations increase the decline in FEV_1_ over time,^22^ and systemic steroid use increases the frequency of complications.^10^ Mepolizumab, one of the available biologic agents for use in severe bronchial asthma, decreases the incidence of exacerbations and reduces steroid use.^11^, ^12^ The administration of a biologic for severe asthma with ICI has been previously reported to be successful in the treatment of EGPA that developed during treatment with nivolumab, an anti‐PD‐1 antibody.^6^ This case also had a different immune status from the usual severe bronchial asthma due to ICIs, but the biologic was effective. In the present cases, mepolizumab was effective; however, other biologics that suppress Th2‐type inflammation, such as benralizumab and dupilumab, may have also been effective. It will be necessary to continue to accumulate case information to investigate the therapeutic effects of all biologics for the treatment of eosinophil‐induced irAE.

In conclusion, two patients with severe bronchial asthma caused by ICI were treated with mepolizumab and their asthma symptoms improved. Treatment with biologics may be effective in severe cases of poorly controlled bronchial asthma during ICI therapy. We have reported these adverse events to the pharmaceutical companies for pharmacovigilance purposes.

## CONFLICT OF INTEREST

None declared.

## AUTHOR CONTRIBUTION

Conceptualization: Toshiyuki Sumi. Investigation: Toshiyuki Sumi, Yuta Nagahisa, Kengo Matsuura, Motoki Sekikawa and Yuichi Yamada. Writing—original draft: Toshiyuki Sumi. Writing—review and editing: Hisashi Nakata and Hirofumi Chiba.

## ETHICS STATEMENT

The authors declare that appropriate written informed consent was obtained for the publication of this case report and accompanying images.

## References

[1] Cai J , Wang D , Zhang G , Guo X . The role of PD‐1/PD‐L1 axis in Treg development and function: implications for cancer immunotherapy. Onco Targets Ther. 2019;12:8437–45. 31686860 10.2147/OTT.S221340 PMC6800566

[2] Bernard‐Tessier A , Jeanville P , Champiat S , Lazarovici J , Voisin AL , Mateus C , et al. Immune‐related eosinophilia induced by anti‐programmed death 1 or death‐ligand 1 antibodies. Eur J Cancer. 2017;81:135–7. 28624693 10.1016/j.ejca.2017.05.017

[3] Scanvion Q , Béné J , Gautier S , Grandvuillemin A , Le Beller C , Chenaf C , et al. Moderate‐to‐severe eosinophilia induced by treatment with immune checkpoint inhibitors: 37 cases from a national reference center for hypereosinophilic syndromes and the French pharmacovigilance database. Oncoimmunology. 2020;9:1722022. 32313716 10.1080/2162402X.2020.1722022 PMC7153834

[4] Maeno K , Fukuda S , Oguri T , Niimi A . Nivolumab‐induced asthma in a patient with non‐small‐cell lung cancer. Ann Oncol. 2017;28:2891. 10.1093/annonc/mdx455 28945837

[5] Donato AA , Krol R . Allergic bronchopulmonary aspergillosis presumably unmasked by PD‐1 inhibition. BMJ Case Rep. 2019;12:e227814. 10.1136/bcr-2018-227814 PMC6381940 30765445

[6] Harada M , Naoi H , Yasuda K , Ito Y , Kagoo N , Kubota T , et al. Programmed cell death‐1 blockade in kidney carcinoma may induce eosinophilic granulomatosis with polyangiitis: a case report. BMC Pulm Med. 2021;21:1–6. 33407304 10.1186/s12890-020-01375-5 PMC7789237

[7] Roger A , Groh M , Lorillon G , Le Pendu C , Maillet J , Arangalage D , et al.; PATIO Group. Eosinophilic granulomatosis with polyangiitis (Churg‐Strauss) induced by immune checkpoint inhibitors. Ann Rheum Dis. 2019;78:e82. 29936437 10.1136/annrheumdis-2018-213857

[8] Skribek M , Rounis K , Afshar S , Grundberg O , Friesland S , Tsakonas G , et al. Effect of corticosteroids on the outcome of patients with advanced non‐small cell lung cancer treated with immune‐checkpoint inhibitors. Eur J Cancer. 2021;145:245–54. 33419647 10.1016/j.ejca.2020.12.012

[9] de La Rochefoucauld J , Noël N , Lambotte O . Management of immune‐related adverse events associated with immune checkpoint inhibitors in cancer patients: a patient‐centred approach. Intern Emerg Med. 2020;15:587–98. 32144552 10.1007/s11739-020-02295-2

[10] Sullivan PW , Ghushchyan VH , Globe G , Schatz M . Oral corticosteroid exposure and adverse effects in asthmatic patients. J Allergy Clin Immunol. 2018;141:110–6. 28456623 10.1016/j.jaci.2017.04.009

[11] Ortega HG , Liu MC , Pavord ID , Brusselle GG , FitzGerald JM , Chetta A , et al.; MENSA Investigators. Mepolizumab treatment in patients with severe eosinophilic asthma. N Engl J Med. 2014;371:1198–207. 25199059 10.1056/NEJMoa1403290

[12] Chupp GL , Bradford ES , Albers FC , Bratton DJ , Wang‐Jairaj J , Nelsen LM , et al. Efficacy of mepolizumab add‐on therapy on health‐related quality of life and markers of asthma control in severe eosinophilic asthma (MUSCA): a randomised, double‐blind, placebo‐controlled, parallel‐group, multicentre, phase 3b trial. Lancet Respir Med. 2017;5:390–400. 28395936 10.1016/S2213-2600(17)30125-X

[13] Wechsler ME , Akuthota P , Jayne D , Khoury P , Klion A , Langford CA , et al. Mepolizumab or placebo for eosinophilic granulomatosis with polyangiitis. N Engl J Med. 2017;376:1921–32. 28514601 10.1056/NEJMoa1702079 PMC5548295

[14] Gupta A , Ikeda M , Geng B , Azmi J , Price RG , Bradford ES , et al. Long‐term safety and pharmacodynamics of mepolizumab in children with severe asthma with an eosinophilic phenotype. J Allergy Clin Immunol. 2019;144:1336–42.e7. 31425781 10.1016/j.jaci.2019.08.005

[15] Moore WC , Kornmann O , Humbert M , Poirier C , Bel EH , Kaneko N , et al. Stopping versus continuing long‐term mepolizumab treatment in severe eosinophilic asthma (COMET study). Eur Respir J. 2021;15:2100396. 10.1183/13993003.00396-2021 PMC8733344 34172470

[16] Akbari O , Stock P , Singh AK , Lombardi V , Lee WL , Freeman GJ , et al. PD‐L1 and PD‐L2 modulate airway inflammation and iNKT‐cell‐dependent airway hyperreactivity in opposing directions. Mucosal Immunol. 2010;3:81–91. 19741598 10.1038/mi.2009.112 PMC2845714

[17] Bratke K , Fritz L , Nokodian F , Geißler K , Garbe K , Lommatzsch M , et al. Differential regulation of PD‐1 and its ligands in allergic asthma. Clin Exp Allergy. 2017;47:1417–25. 28865147 10.1111/cea.13017

[18] Kubo S , Yamada T , Osawa Y , Ito Y , Narita N , Fujieda S . Cytosine‐phosphate‐guanosine‐DNA induces CD274 expression in human B cells and suppresses T helper type 2 cytokine production in pollen antigen‐stimulated CD4‐positive cells. Clin Exp Immunol. 2012;169:1–9. 22670772 10.1111/j.1365-2249.2012.04585.x PMC3390467

[19] Nasiri Kalmarzi R , Fattahi N , Kaviani Z , Ataee P , Mansouri M , Moradi G , et al. Inverse correlation of soluble programmed cell death‐1 ligand‐1 (sPD‐L1) with eosinophil count and clinical severity in allergic rhinitis patients. Allergol Int. 2017;66:326–31. 27617656 10.1016/j.alit.2016.08.008

[20] Varricchi G , Galdiero MR , Loffredo S , Lucarini V , Marone G , Mattei F , et al. Eosinophils: the unsung heroes in cancer? Oncoimmunology. 2017;7:e1393134. 29308325 10.1080/2162402X.2017.1393134 PMC5749653

[21] Krishnan T , Tomita Y , Roberts‐Thomson R . A retrospective analysis of eosinophilia as a predictive marker of response and toxicity to cancer immunotherapy. Future Sci OA. 2020;6:FSO608. 33312694 10.2144/fsoa-2020-0070 PMC7720365

[22] Matsunaga K , Hirano T , Oka A , Tanaka A , Kanai K , Kikuchi T , et al. Progression of irreversible airflow limitation in asthma: correlation with severe exacerbations. J Allergy Clin Immunol Pract. 2015;3:759–64. 26054551 10.1016/j.jaip.2015.05.005

